# Dietary Epidermal Growth Factor Supplementation Alleviates Intestinal Injury in Piglets with Intrauterine Growth Retardation via Reducing Oxidative Stress and Enhancing Intestinal Glucose Transport and Barrier Function

**DOI:** 10.3390/ani12172245

**Published:** 2022-08-30

**Authors:** Xiaopeng Tang, Kangning Xiong

**Affiliations:** State Engineering Technology Institute for Karst Desertfication Control, School of Karst Science, Guizhou Normal University, No. 116 North Baoshan Road, Yunyan District, Guiyang 550001, China

**Keywords:** epidermal growth factor, glucose absorption, intestinal barrier function, intrauterine growth retardation, oxidative stress, piglets

## Abstract

**Simple Summary:**

It is well known that intrauterine growth retardation (IUGR) seriously affects the postnatal growth and intestinal health of piglets. Keeping intestinal health is of great importance to pig production. Numerous studies have shown that epidermal growth factor (EGF) has a positive effect on the repair of intestinal injury of animals. However, whether EGF can promote the intestinal health of IUGR piglets is unknown. Therefore, in our present study, a total of 12 IUGR piglets and 6 normal birth weight (NBW) piglets were selected to study the effects of EGF on the growth performance and intestinal health of IUGR piglets. Our study found that basal diet supplemented with 2 mg/kg EGF significantly improved the growth performance, jejunum morphology, jejunum glucose absorption and antioxidant capacity, and jejunum barrier function of piglets with IUGR. Therefore, this study confirmed that EGF has positive effects on the growth and intestinal development of piglets with IUGR.

**Abstract:**

EGF plays an important role in the intestinal repair and nutrients transport of animals. However, the effect of EGF on the intestinal health of piglets with IUGR has not been reported. Thus, the present study was performed to investigate the effects of EGF on the intestinal morphology, glucose absorption, antioxidant capacity, and barrier function of piglets with IUGR. A total of 6 NBW piglets and 12 IUGR piglets were randomly divided into three treatments: NC group (NBW piglets fed with basal diet, *n* = 6), IC group (IUGR piglets fed with basal diet, *n* = 6), and IE group (IUGR piglets fed with basal diet supplemented with 2 mg/kg EGF, *n* = 6). Growth performance, serum biochemical profile, jejunum histomorphology, jejunum glucose absorption and antioxidant capacity, and jejunal barrier function were measured. The results showed that EGF supplementation significantly increased the final body weight (FBW), average daily gain (ADG), and average daily feed intake (ADFI) of piglets with IUGR; EGF supplementation significantly increased the total protein (TP), glucose (GLU), and immunoglobulin G (IgG) levels compared with the IUGR piglets in the IC group; EGF administration effectively exhibited an increased jejunum villus height (VH) and the villus-height-to-crypt-depth ratio (V/C) of IUGR piglets compared with the IC group; EGF supplementation significantly increased sodium/potassium-transporting adenosine triphosphatase (Na^+^/K^+^-ATPase) activity, intestinal alkaline phosphatase (AKP) activity, glucose transporter sodium/glucose cotransporter 1 (*SGLT1*), glucose transporter 2 (*GLUT2*), and AMP-activated protein kinase α1 (*AMPK-α1*) mRNA expressions in the jejunum of IUGR piglets compared with the IC group; EGF supplementation exhibited increased superoxide dismutase (SOD) activity and total antioxidant capacity (T-AOC) levels, tended to increase glutathione peroxidase (GSH-Px) and catalase (CAT) activities, and tended to decrease the malondialdehyde (MDA) level in the jejunum of IUGR piglets compared with the IC group; EGF supplementation significantly increased *ZO-1*, *Claudin-1*, *Occludin*, and *MUC2* mRNA expressions and improved secreted immunoglobulin A (sIgA) secretion in the jejunum of IUGR piglets compared with the IC group and tended to decrease the interleukin 1β (IL-1β), IL-6, and tumor necrosis factor α (TNF-α) levels in the jejunum of IUGR piglets compared with the IC group. Pearson’s correlation analysis further showed that EGF can promote intestinal development and nutrient absorption by promoting intestinal barrier function, thus improving the growth performance of IUGR piglets.

## 1. Introduction

IUGR usually refers to impaired growth and development of the embryo and/or its organs during pregnancy [1,2]. Pigs, a kind of multifetal mammal, exhibit a high incidence of IUGR, which seriously affects the postnatal growth and intestinal health of piglets and ultimately affects the economic benefit of pig production [3,4]. Studies have confirmed that piglets with IUGR are associated with feeding intolerance and intestinal dysfunction [4,5], which leads to a retard growth and poor health status of pigs. Therefore, strategies to alleviate intestinal injury and promote intestinal development may help to reverse the growth retardation in IUGR pigs. Nowadays, many strategies have been tested to reduce the incidence of IUGR at the sow level [6,7] or through nutritional interventions at the piglet level to increase IUGR piglets’ neonatal survival and growth [8,9].

The intestinal tract is an important site for the digestion and absorption of nutrients, and also functions as an important barrier to prevent the entry of an exogenous harmful substance into the circulation system [10,11]. Thus, maintaining a healthy gut is crucial for an animal’s overall health. IUGR is associated with intestinal injury of piglets, which can be demonstrated by the impaired intestinal morphology, raised apoptosis of intestinal epithelial cells, and increased oxidative injury and inflammatory response [4,8,12,13,14]. Emerging evidence suggests that EGF is a promising therapeutic treatment for intestinal injury due to its multiple biological functions [15,16,17,18,19]. EGF exhibits potential antioxidant and anti-inflammatory activities, and it can protect enterocytes against alcohol-induced inflammation [20], hydrogen peroxide [19] and acetaldehyde [21] caused barrier dysfunction, and lipopolysaccharide (LPS) [16] and ischemia/reperfusion [22]-induced oxidative injury. However, there are little data on EGF as a nutritional additive to alleviate intestinal injury in IUGR offspring. Our previous study showed that EGF treatment can promote glucose absorption of damaged intestinal or intestinal epithelial cells of piglets [18,23], which means that glucose plays an important role in intestinal repair. However, whether EGF can promote intestinal repair of IUGR piglets by increasing intestinal glucose absorption has not been reported. Therefore, it has a certain application value to study the effect of EGF on the intestinal health of IUGR piglets.

Accordingly, we hypothesized that dietary EGF supplementation can alleviate intestinal injury of piglets with IUGR by reducing intestinal oxidative stress and enhancing intestinal glucose transport and barrier function. To test the above hypothesis, we studied the effects of EGF on intestinal oxidative damage, barrier function, and the glucose absorption capacity of piglets with IUGR. This research can provide a reference for the application of EGF in IUGR piglets and provide some reliable reference for intestinal disorders treating in IUGR human neonates.

## 2. Materials and Methods

### 2.1. Animals and Experimental Design

In this study, 6 NBW piglets (1.55 ± 0.05 kg; within one SD of the mean birth weight) and 12 IUGR piglets (0.94 ± 0.06; two SD below the mean birth weight) were selected from 6 sows with similar parity. All piglets suckled by their mother sows. At weaning (21 days of age), 6 NBW piglets were assigned to the NC group (NBW piglets fed with basal diet, *n* = 6), and 12 IUGR piglets were randomly divided into the IC group (IUGR piglets fed with basal diet, *n* = 6) and the IE group (IUGR piglets fed with basal diet supplemented with 2 mg/kg EGF, *n* = 6). The EGF product (pig source) was obtained from Zyme Fast (Changsha, China) Biotechnology (Changsha, China) with egg yolk powder as the carrier, and its purity was 4000 mg/kg. The composition and nutrient levels of basal diet (Table 1) met the nutrient specifications for 5 to 10 kg BW pig according to the NRC (2012) [24]. The experimental piglets were housed individually and had free access to feed and water. The room temperature was maintained at an ambient temperature range of 25–28 °C, and the room lighting was natural. The experiment lasted for 14 days.

The body weights of piglets were weighed at the beginning and the end of the animal experiment to calculate the ADG of piglets. The feed intake was recorded weekly to calculate the ADFI of piglets. The feed-to-gain ratio (F/G) of piglets was calculated by ADFI/ADG [25].

### 2.2. Sample Collection

All animals were fasted for 12 h before sampling. All piglets were anesthetized by injection of 50 mg kg^−1^ BW sodium pentobarbital, and 10 mL of blood was collected from the jugular vein and then centrifuged at 3000× *g* for 15 min at 4 °C to obtain serum samples. After blood collection, all pigs were killed by exsanguinations to death, after which the abdomens of the piglets were quickly opened for the removal of the entire intestine to isolate the jejunum. The middle jejunum segment (approximately 2 cm) was fixed in 10% neutral buffered formalin for intestinal morphology measurement. The intestinal mucosa of the middle jejunum tissue was gently scraped down and then rapidly frozen in liquid nitrogen and stored at −80 °C for subsequent analysis.

### 2.3. Serum Biochemical Profile Detection

Serum alanine aminotransferase (ALT), aspartate aminotransferase (AST), urea nitrogen (BUN), TP, GLU, T), immunoglobulin A (IgA), IgG, and IgM were measured using an automatic blood analyzer, Mindray BS-420 (Shenzhen Mindray Bio-Medical Electronics Co., Ltd., Shenzhen, China), in accordance with the manufacturer’s instructions.

### 2.4. Intestinal Histomorphology

Jejunum samples were fixed in 10% neutral buffered formalin for 24 h, then dehydrated and embedded in paraffin. Specimens were sliced into a thickness of 4 μm and stained with hematoxylin and eosin for morphological evaluation. A light microscope with a computer-assisted morphometric system (BioScan Optimetric, BioScan Inc., Edmonds, WA, USA) was used to measure VH and crypt depth (CD) [26]. V/C was calculated by VH/CD.

### 2.5. Jejunal Mucosal Antioxidant and Immune Indices Detection

Jejunal mucosa samples (about 250 mg) were homogenized with a high-speed benchtop homogenizer (Tekmar, Cincinnati, OH, USA) in saline solution (1:4, weight/volume), then centrifuged at 3500 r/min for 15 min at 4 °C. The supernatants were collected for subsequent analysis. The MDA content was detected by thiobarbituric acid (TBA) method (kit no. A003-1-2), GSH-Px activity was detected by measuring the consumption of reduced glutathione in enzymatic reaction (kit no. A005-1-2), SOD activity was detected by hydroxylamine method (kit no. A001-1-2), CAT activity was detected by ammonium molybdate method (kit no. A007-1-1), and trolox equivalents were used for T-AOC measured (kit no. A015-2-1). All kits were purchased from Nanjing Jiancheng Bioengineering Institute (Nanjing, China). sIgA (kit no. CSB-E12063p), IL-1β (kit no. CSB-E09725p), IL-6 (kit no. CSB-E06786p), and TNF-α (kit no. CSB-E16980p) were measured using porcine-specific enzyme-linked immunosorbent assay (ELISA) kits according to the manufacturer’s instructions (Cusabio Biotech Co., Wuhan, China).

### 2.6. AKP and Na^+^/K^+^-ATPase Activity in Jejunum Mucosa

ATPase can decompose ATP to produce ADP and inorganic phosphorus, and the Na^+^/K^+^-ATPase activity can be judged by measuring the amount of inorganic phosphate. The intestinal Na^+^/K^+^-ATPase activity (kit no. A070-2-2) was measured using a commercial kit purchased from Nanjing Jiancheng Bioengineering Institute (Nanjing, China). AKP can decompose benzene disodium phosphate to produce free phenol and phosphoric acid. The phenol reacts with 4-aminoantipyrine in alkaline solution and oxidizes to red quinone derivatives by potassium ferricyanide. The AKP activity can be measured according to the red color. The intestinal AKP activity (kit no. A059-2-2) was measured using a commercial kit purchased from Nanjing Jiancheng Bioengineering Institute (Nanjing, China).

### 2.7. Real-Time PCR

Total RNA extraction and reverse transcription were performed as described previously [27]. The primers used in this study (Table 2) were synthesized by Sangon Biotech (Shanghai, China). The formula was 2^−^^(ΔΔCt)^, where ΔΔCt = (Ct_Target_ − Ct_β-actin_) treatment − (Ct_Target_ − Ct_β-actin_) control was used to calculated the relative gene expression [4].

### 2.8. Statistical Analysis

Data were presented as the mean ± SEM. Data analysis was performed by one-way ANOVA procedure of SPSS Statistics 21.0 (SPSS, Inc., Chicago, IL, USA). Differences among treatment mean were determined using a Tukey multiple comparison test procedure of SPSS Statistics 21.0 (SPSS, Inc., Chicago, IL, USA). The results of all data analyses were input into the GraphPad Prism 9.0 (GraphPad Software, Inc., San Diego, CA, USA) software for graphical display [4]. The correlations analysis was performed by the genescloud tools, a free online platform for data analysis (https://www.genescloud.cn (accessed on 25 June 2022)). *p* < 0.05 was considered to be statistically significant.

## 3. Results

### 3.1. Growth Performance 

As shown in Table 3, the initial body weight (IBW), FBW, ADG, and ADFI were significantly different among three treatments (*p* < 0.01). Compared with the NC group, the IC group had a lower IBW, FBW, ADG, and ADFI (*p* < 0.05) during the first 2 weeks after weaning. Compared with the IC group, the IE group had a higher (*p* < 0.05) FBW, ADG, and ADFI, although significantly lower (*p* < 0.05) than the CN group. 

### 3.2. Serum Biochemical Profiles

As shown in Table 4, the concentrations of serum AST, TP, GLU, IgG, and IgM were markedly changed in this study (*p* < 0.05). The IC group had a higher (*p* < 0.05) serum AST level and lower (*p* < 0.05) TP, GLU, IgG, and IgM levels compared with the NC group. EGF supplementation significantly increased (*p* < 0.05) the TP, GLU, and IgG levels of IUGR piglets compared with the IC group and showed no difference compared with the NC group.

### 3.3. Intestinal Morphology and Its Correlations Analysis with Growth Performance

**Figure 1:**
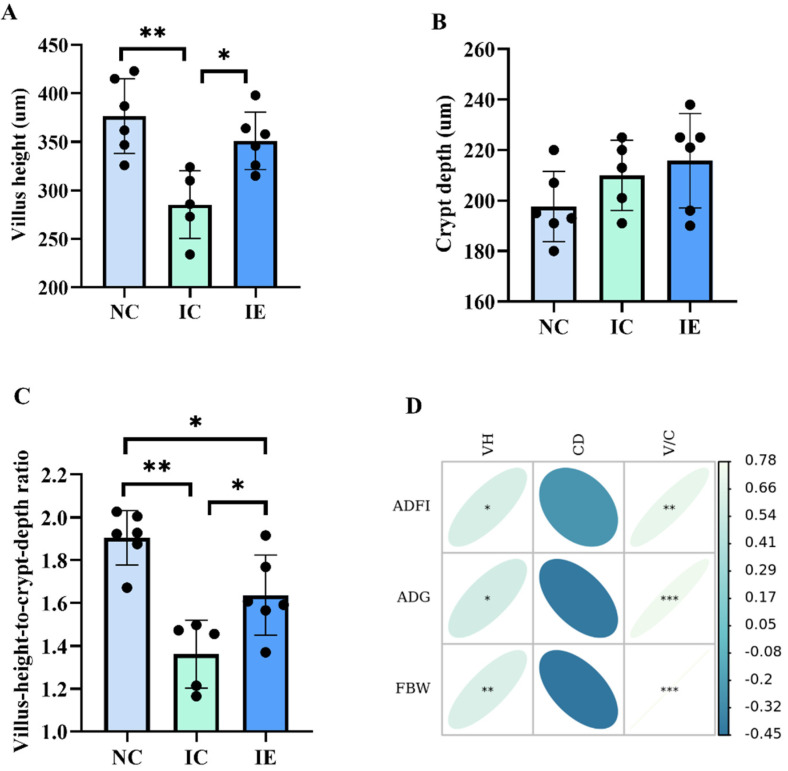
Effects of EGF on the jejunum morphology of piglets with IUGR. (**A**) villus height, (**B**) crypt depth, (**C**) villus height to crypt depth, (**D**) correlations analysis between intestinal morphology and growth performance. Values are expressed as means ± SEM, *n* = 6 (one pig in the IC group was dead, *n* = 5 in IC group); NC: NBW piglets fed with basal diet; IC: IUGR piglets fed with basal diet; IE: IUGR piglets fed with basal diet supplemented with 2 mg/kg EGF. * *p* < 0.05, ** *p* < 0.01, *** *p* < 0.001.

The effects of EGF on the intestinal morphology of piglets with IUGR are presented in Figure 1. It shows that VH (Figure 1A) and V/C (Figure 1C) were markedly changed in this study (*p* < 0.05); however, there was no difference in CD (Figure 1B) among the three groups. IUGR reduces VH (*p* < 0.01) and V/C (*p* < 0.01) significantly. Meanwhile, EGF administration exhibited increased (*p* < 0.05) VH and V/C in the jejunum of IUGR piglets (IE group) when compared with the IC group. Pearson’s correlation analysis (Figure 1D) showed that VH (*p* < 0.05, *p* < 0.05, *p* < 0.01) and V/C (*p* < 0.01, *p* < 0.001, *p* < 0.001) were positively correlated with the ADFI, ADG, and FBW of piglets.

### 3.4. Glucose Absorption Capacity and Its Correlation Analysis with Growth Performance and Intestinal Morphology 

**Figure 2:**
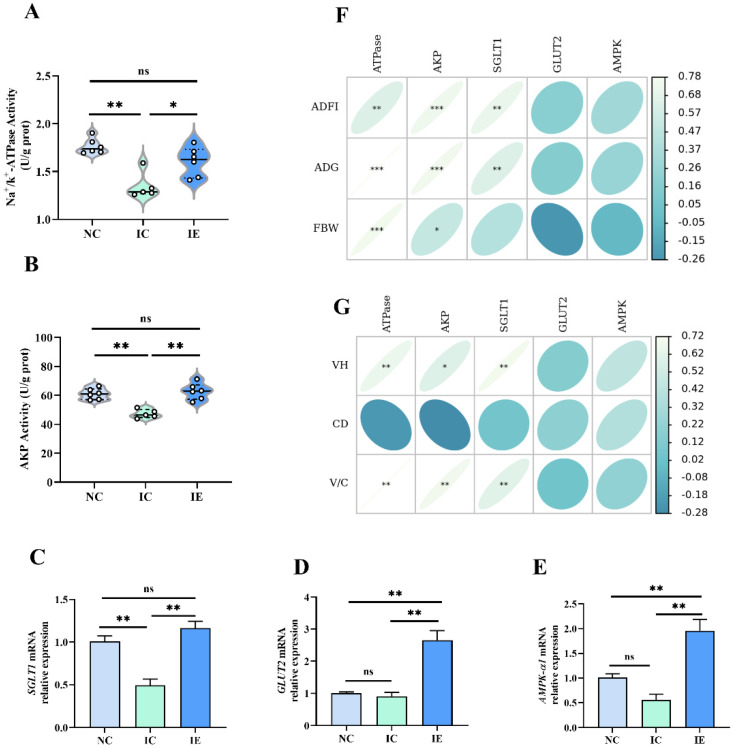
Effects of EGF on the intestinal glucose absorption capacity of piglets with IUGR. (**A**) Na^+^/K^+^-ATPase activity, (**B**) AKP activity, (**C**) *SGLT1* mRNA, (**D**) *GLU2* mRNA, (**E**) *AMPK-α1* mRNA, (**F**) correlations analysis between intestinal glucose absorption capacity and growth performance, (**G**) correlations analysis between intestinal glucose absorption capacity and intestinal morphology. Values are expressed as means ± SEM, *n* = 6 (1 pig in the IC group was dead, *n* = 5 in the IC group); NC: NBW piglets fed with basal diet; IC: IUGR piglets fed with basal diet; IE: IUGR piglets fed with basal diet supplemented with 2 mg/kg EGF. * *p* < 0.05, ** *p* < 0.01, *** *p* < 0.001.

The effects of EGF on the intestinal glucose absorption capacity of piglets with IUGR are presented in Figure 2. The activities of Na^+^/K^+^-ATPase activity (Figure 2A) and AKP activity (Figure 2B) and the gene expression of *SGLT1* (Figure 2C), *GLUT2* (Figure 2D), and *AMPK-α1* (Figure 2E) were significantly different among the three treatments (*p* < 0.05). The IUGR piglets in the IC group had a lower intestinal Na^+^/K^+^-ATPase activity (*p* < 0.01), AKP activity (*p* < 0.01), and *SGLT1* mRNA expression (*p* < 0.01;) compared with the NBW piglets in the NC group; IUGR did not affect *GLUT2* and *AMPK-α1* mRNA expression. EGF supplementation significantly increased Na^+^/K^+^-ATPase activity (*p* < 0.05), AKP activity (*p* < 0.01), *SGLT1* (*p* < 0.01), *GLUT2* (*p* < 0.01), and *AMPK-α1* (*p* < 0.01) mRNA expression in the jejunum of IUGR piglets compared with IUGR piglets without EGF supplementation. Compared with the NC group, IUGR piglets in the IE group had a higher (*p* < 0.01) *GLUT2* and *AMPK-α1* mRNA expression, and there was no difference in Na^+^/K^+^-ATPase activity, AKP activity, and *SGLT1* mRNA expression between the IE group and the NC group. Pearson’s correlation analysis showed that the Na^+^/K^+^-ATPase activity (*p* < 0.01, *p* < 0.001; *p* < 0.001) and AKP activity (*p* < 0.001, *p* < 0.001; *p* < 0.05) were positively correlated with the ADFI, ADG, and FBW of piglets; and *SGLT1* gene expression (*p* < 0.01, *p* < 0.01) was positively correlated with the ADFI and ADG of piglets (Figure 2F). Na^+^/K^+^-ATPase activity (*p* < 0.01, *p* < 0.01), AKP activity (*p* < 0.05, *p* < 0.01), and *SGLT1* mRNA expression (*p* < 0.01, *p* < 0.01) were positively correlated with VH and V/C (Figure 2G).

### 3.5. Antioxidant Activities and Its Correlation Analysis with Growth Performance and Intestinal Morphology

**Figure 3:**
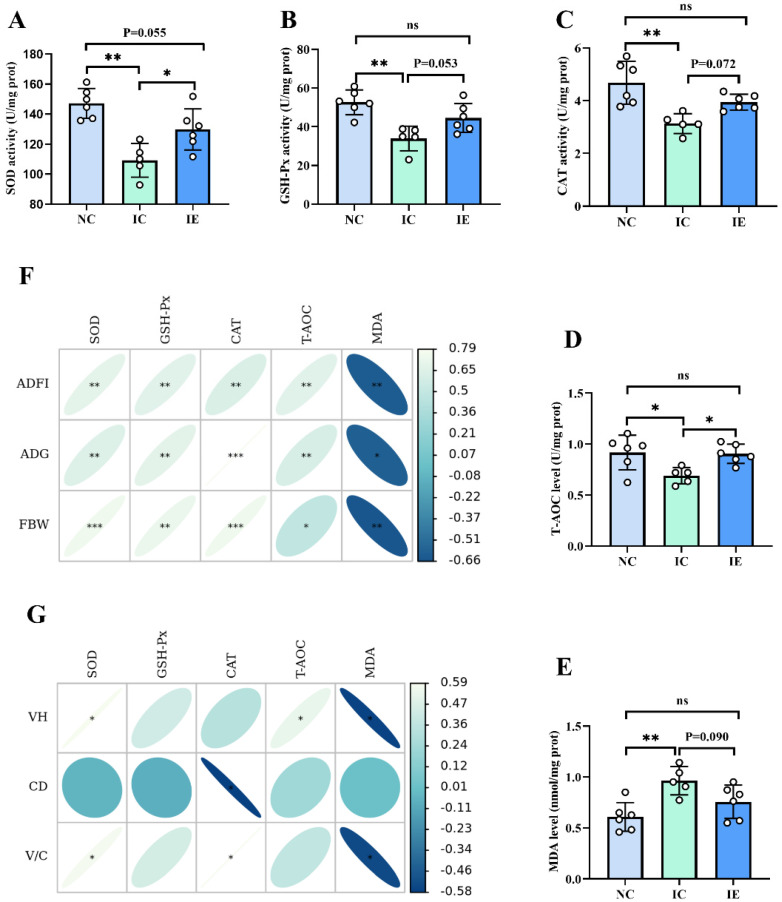
Effects of EGF on the intestinal antioxidant capacity of piglets with IUGR. (**A**) SOD, (**B**) GSH-Px, (**C**) CAT, (**D**) T-AOC, (**E**) MDA, (**F**) correlation analysis between intestinal antioxidant capacity and growth performance, (**G**) correlation analysis between intestinal antioxidant capacity and intestinal morphology. Values are expressed as means ± SEM, *n* = 6 (1 pig in the IC group was dead, *n* = 5 in the IC group); NC: NBW piglets fed with basal diet; IC: IUGR piglets fed with basal diet; IE: IUGR piglets fed with basal diet supplemented with 2 mg/kg EGF. * *p* < 0.05, ** *p* < 0.01, *** *p* < 0.001.

As shown in Figure 3, there was a significant difference (*p* < 0.05) of SOD activity (Figure 3A), GSH-Px activity (Figure 3B), CAT activity (Figure 3C), T-AOC level (Figure 3D), and MDA level (Figure 3E) among the NC, IC, and IE groups. It showed that the IC group had a lower SOD activity (*p* < 0.01), GSH-Px activity (*p* < 0.01), CAT activity (*p* < 0.01), and T-AOC level (*p* < 0.05) and a higher MDA level (*p* < 0.01) compared with the NC group. The IE group exhibited an increased SOD activity (*p* < 0.05) and T-AOC level (*p* < 0.05) compared with the IC group; EGF supplementation tended to increase the GSH-Px activity (*p* = 0.053) and CAT activity (*p* = 0.072) and tended to decrease the MDA level (*p* = 0.090) in the jejunum of IUGR piglets compared with IUGR piglets without EGF supplementation. No differences in GSH-Px, SOD, CAT, T-AOC, and MDA were observed between the IE group and the NC group. Pearson’s correlation analysis showed that SOD activity (*p* < 0.01, *p* < 0.01, *p* < 0.001), GSH-Px activity (*p* < 0.01, *p* < 0.01, *p* < 0.01), CAT activity (*p* < 0.01, *p* < 0.001, *p* < 0.001), and T-AOC level (*p* < 0.01, *p* < 0.01, *p* < 0.05) were positively correlated with the ADFI, ADG, and FBW of piglets, and the MDA level (*p* < 0.01, *p* < 0.05, *p* < 0.01) was negatively correlated with the ADFI, ADG, and FBW of piglets (Figure 3F). SOD activity was positively correlated with VH and V/C (*p* < 0.05), CAT activity was positively correlated with V/C (*p* < 0.05) and negatively correlated with CD (*p* < 0.05), T-AOC level was positively correlated with VH (*p* < 0.05), and MDA level (*p* < 0.05) was negatively correlated with VH and V/C (Figure 3G).

### 3.6. Intestinal Barrier Function and Its Correlation Analysis with Growth Performance and Intestinal Morphology

**Figure 4:**
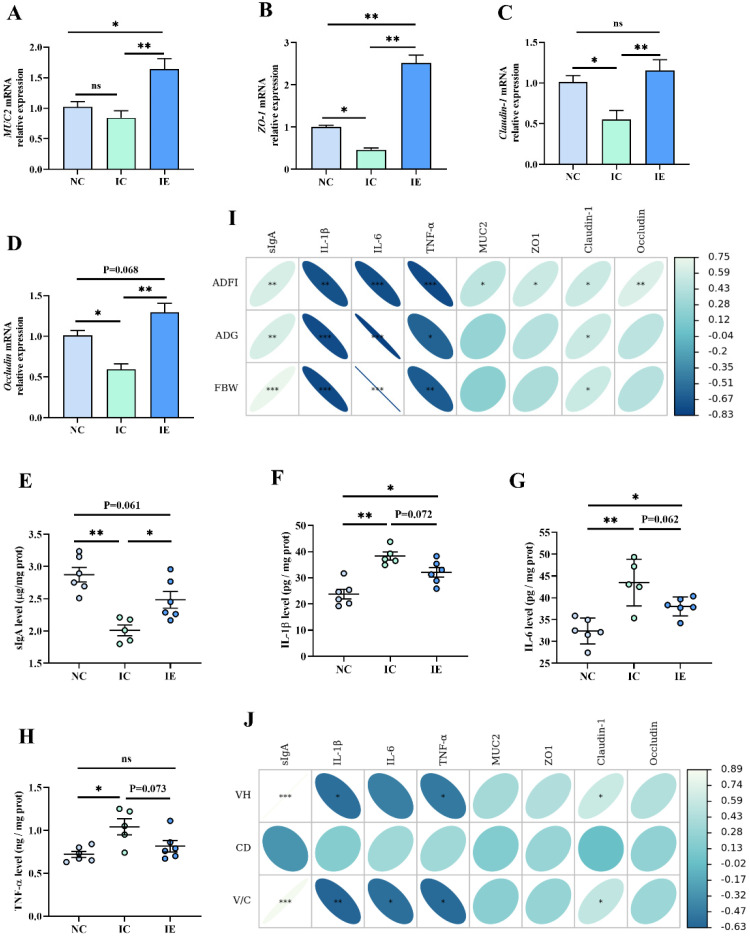
Effects of EGF on the intestinal barrier function of piglets with IUGR. (**A**) *MUC2* mRNA, (**B**) *ZO-1* mRNA, (**C**) *Claudin-1* mRNA, (**D**) *Occludin* mRNA, (**E**) sIgA level, (**F**) IL-1β level, (**G**) IL-6 level, (**H**) TNF-α level, (**I**) correlation analysis between intestinal barrier function and growth performance, and (**J**) correlation analysis between barrier function and intestinal morphology. Values are expressed as means ± SEM, *n* = 6 (1 pig in the IC group was dead, *n* = 5 in the IC group); NC: NBW piglets fed with basal diet; IC: IUGR piglets fed with basal diet; IE: IUGR piglets fed with basal diet supplemented with 2 mg/kg EGF. * *p* < 0.05, ** *p* < 0.01, *** *p* < 0.001.

As shown in Figure 4, *MUC2* (Figure 4A), *ZO-1* (Figure 4B), *Claudin-1* (Figure 4C), and *Occludin* (Figure 4D) mRNA expression and the secretion of sIgA (Figure 4E), IL-1β (Figure 4F), IL-6 (Figure 4G), and TNF-α (Figure 4H) were significantly different among the three treatments (*p* < 0.05). Results showed that IUGR did not affect the *MUC2* mRNA expression in jejunum mucosa. The IC group had a lower (*p* < 0.05) *ZO-1*, *Claudin-1*, and *Occludin* gene expression and a lower sIgA (*p* < 0.01) secretion in jejunum mucosa compared with the IC group; the IC group had a higher IL-1β (*p* < 0.01), IL-6 (*p* < 0.01), and TNF-α level (*p* < 0.05) in jejunum mucosa compared with the IC group. EGF supplementation (IE group) significantly (*p* < 0.01) increased the *ZO-1*, *Claudin-1*, *Occludin*, and *MUC2* mRNA expression and improved the sIgA secretion in jejunum mucosa compared with the IC group, and significantly increased *ZO-1* (*p* < 0.01) and *MUC2* (*p* < 0.05) mRNA expression compared with the NC group, and tended to decrease the IL-1β (*p* = 0.072), IL-6 (*p* = 0.062), and TNF-α level (*p* = 0.073) in jejunum mucosa compared with the IC group. Pearson’s correlation analysis showed that the *ZO-1* (*p* < 0.05), *MUC2* (*p* < 0.05), and *Occludin* (*p* < 0.01) gene expressions were positively correlated with the ADFI of piglets; the *Claudin-1* gene expression (*p* < 0.05, *p* < 0.05, *p* < 0.05) and sIgA (*p* < 0.01, *p* < 0.01, *p* < 0.001) were positively correlated with the ADFI, ADG, and the FBW of piglets; and TNF-α (*p* < 0.01, *p* < 0.05, *p* < 0.01), IL-6 (*p* < 0.001, *p* < 0.001, *p* < 0.001), and IL-1 level (*p* < 0.01, *p* < 0.001, *p* < 0.001) were negatively correlated with the ADFI, ADG, and the FBW of piglets (Figure 4I). *Claudin-1* gene expression (*p* < 0.05, *p* < 0.05) and sIgA (*p* < 0.001, *p* < 0.001) were positively correlated with VH and V/C, and the TNF-α (*p* < 0.05, *p* < 0.05) and IL-1 level (*p* < 0.05, *p* < 0.05) were negatively correlated with VH and V/C, and the IL-6 level (*p* < 0.05) was negatively correlated with V/C (Figure 4J).

### 3.7. The Correlation Analysis of Intestinal Barrier Function with Glucose Absorption Capacity

**Figure 5:**
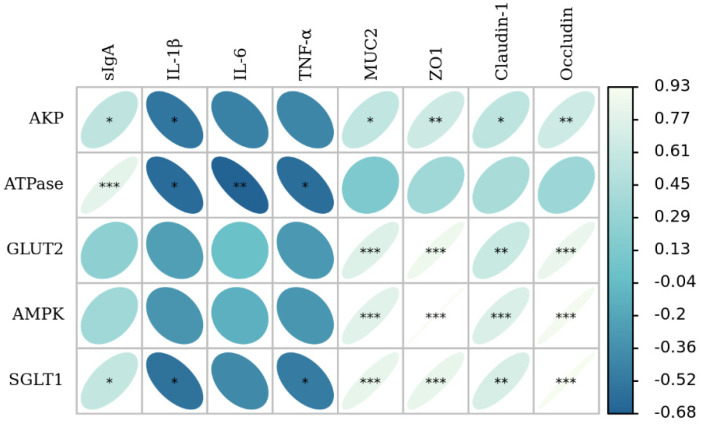
Correlation analysis between intestinal barrier function and intestinal glucose absorption capacity. * *p* < 0.05, ** *p* < 0.01, *** *p* < 0.001.

Pearson’s correlation analysis showed that *MUC2* (*p* < 0.05, *p* < 0.001, *p* < 0.001, *p* < 0.001), *ZO-1* (*p* < 0.01, *p* < 0.001, *p* < 0.001, *p* < 0.001), *Claudin-1* (*p* < 0.05, *p* < 0.01, *p* < 0.001, *p* < 0.01), and *Occludin* (*p* < 0.01, *p* < 0.001, *p* < 0.001, *p* < 0.001) gene expression were positively correlated with AKP activity, *GLUT2*, *AMPK α1*, and *SGLT1* mRNA expression; sIgA (*p* < 0.05, *p* < 0.001, *p* < 0.01) was positively correlated with AKP activity, Na^+^/K^+^-ATPase activity, and the gene expression of *SGLT1*; and TNF-α (*p* < 0.05, *p* < 0.05) was negatively correlated with Na^+^/K^+^-ATPase activity and the gene expression of *SGLT1*; the IL-6 level (*p* < 0.01) was negatively correlated with Na^+^/K^+^-ATPase activity; and the IL-1β level (*p* < 0.05, *p* < 0.05, *p* < 0.05) was negatively correlated with AKP activity, Na^+^/K^+^-ATPase activity, and the gene expression of *SGLT1* (Figure 5).

## 4. Discussion

### 4.1. Effects of EGF on the Growth Performance of IUGR Piglets 

Animals with IUGR are characterized by low birth weight and postnatal growth retardation [4,29]. Our previous study showed that IUGR significantly reduced the average body weight gain of piglets in the whole sucking period [4]. In this study, IUGR piglets showed a lower ADG and ADFI compared with the NBW pigs, which was consistent with previous studies [9,30]. EGF has a positive effect on the growth performance of weaned piglets to some extent [17,31], but whether EGF could promote the growth performance of IUGR piglets has not been reported yet. In this study, EGF supplementation significantly increased the FBW, ADG, and ADFI of IUGR piglets compared with IUGR piglets without EGF supplementation. These results indicate that EGF has a certain effect on the alleviating growth inhibition of weaned piglets caused by IUGR.

### 4.2. Effects of EGF on the Serum Biochemical Indices of Piglets with IUGR

Serum biochemical indexes are important indexes reflecting the metabolic status of animals [25,26]. For example, the measurement of the serum activities of the hepatic enzymes ALT and AST was considered a reliable indicator to evaluate the liver function of animals [32]. The serum TP level can reflect the status of protein synthesis and metabolism [33]. Glucose is essential for maintaining the energy and health status of animals and regulating the absorption and transport capacity of various nutrients [18,23]. Immunoglobulins (IgA, IgG, and IgM) play important roles in immunity, which are considered important indicators reflecting the health status of animals [34,35]. In the present study, the AST, TP, Glu, IgA, and IgM indexes were changed remarkably in response to piglets fed with EGF, which means that EGF is beneficial to the growth, nutrient absorption, immunity, protein synthesis, and body health of piglets with IUGR.

### 4.3. Effects of EGF on the Intestinal Morphology of IUGR Piglets

The integrity of the intestinal tract provides a guarantee for the utilization of nutrients [4]. Intestinal VH, CD, and V/C can reflect the healthy state of intestinal function, the decreased CD, and increased VH and V/C of intestines indicate a better intestinal function of animals [36]. Studies have shown that IUGR can cause intestinal villus shedding, crypt hyperplasia, and intestinal mucosal atrophy and damage intestinal mucosal barrier function, thereby resulting in the decline of intestinal absorption function [4,9]. EGF, as an intestinal trophic factor, plays important roles in intestinal development and intestinal repair [15,16,23]. The results of this study showed that dietary EGF supplementation significantly increased VH and V/C, which indicated that EGF could repair the intestinal morphology of piglets with IUGR. Similarly, our previous study showed that EGF treatment could repair intestinal morphology in LPS-stimulated piglets [23]. Pearson’s correlation analysis showed that VH and V/C were positively correlated with the ADFI, ADG, and FBW of piglets, which means that a good intestinal morphology is essential for animal growth.

### 4.4. Effects of EGF on the Glucose Absorption Capacity of Piglets with IUGR

Glucose is an important energy source in the body and is absorbed mainly in the small intestine [36]. It has been proved that the glucose absorption in the intestinal tract is achieved through either the paracellular or transcellular route, in which the Na^+^/glucose cotransporter (SGLT1)-mediated transcellular route is the major route for the glucose transport [4,23,37,38]. GLUT2, a facilitated Na^+^-independent monosaccharide transporter, also contributes to glucose absorption; it can be recruited transiently to the apical membrane and assist in glucose absorption in response to a sugar-rich meal [39] or intestinal repair process [23]. Therefore, the factors that influence SGLT1 and GLUT2 expression would also affect glucose absorption and metabolism. The transmembrane Na^+^ gradient and the membrane potential generated by Na^+^/K^+^-ATPase are the driving forces of SGLT1-mediated glucose transport [40]. AKP speeds up the absorption and transfer of nutrients, indirectly providing energy to the body [23]. It has been shown that sustained impairment of intestinal development in piglets results in decreased intestinal glucose absorption capacity [4,23]. The present study showed that IUGR significantly decreased Na^+^/K^+^-ATPase and AKP activity and *SGLT1* gene expression compared with the NC group, which indicated that IUGR significantly impaired the intestinal glucose absorption capacity of piglets during the weaning period. Dietary EGF supplementation significantly increased Na^+^/K^+^-ATPase and AKP activity and *SGLT1*, *GLUT2*, and *AMPK-α1* mRNA expression compared with the IC group, which demonstrated that EGF can improve the glucose absorption capacity of the damaged intestine caused by IUGR. Our previous studies demonstrated that EGF can promote the glucose absorption capacity of injured IPEC-J2 and the jejunum of piglets induced by LPS [18,23]. Pearson’s correlation analysis showed that glucose absorption capacity was closely related to the intestinal morphology and growth performance of piglets. Thus, this study suggested that one way of EGF to alleviate intestinal injury and promote the growth performance of IUGR piglets is by improving intestinal glucose absorption capacity.

### 4.5. Effects of EGF on the Antioxidant Activities of Piglets with IUGR

An increased MDA level and decreased SOD, CAT, and GSH-Px levels are generally considered markers of intestinal oxidative injury [4,16,41]. Substantial evidence indicates that IUGR impairs the antioxidant defense system by increasing MDA production and decreasing antioxidant enzyme activity [4,8,13,42]. Similarly, in the present study, the IC group had a higher MDA content and lower GSH-Px, SOD, CAT, and T-AOC levels than the CN group, which indicated that IUGR results in jejunum oxidative damage in piglets during the first 2 weeks after weaning. EGF is an important cell protective peptide, which has certain antioxidant function and can alleviate oxidative injury induced by LPS [16] and intestinal ischemia/reperfusion [22]. Our previous study showed that EGF exhibited potent protective effects on IPEC-J2 cells against LPS-induced cell oxidative damage by enhancing GSH-Px, SOD, and CAT expressions [16]. In this study, the addition of EGF significantly increased SOD activity and T-AOC level, tended to increase GSH-Px and CAT activity, and tended to decrease MDA level compared with the IC group, which indicated that EGF could alleviate intestinal oxidative damage of IUGR piglets. Pearson’s correlation analysis showed that intestinal redox status was closely related to the growth performance and intestinal morphology of piglets. Thus, the results of this study indicated that EGF can improve growth performance and alleviate the intestinal injury of IUGR piglets by enhancing intestinal antioxidant capacity.

### 4.6. Effects of EGF on the Intestinal Barrier Function of Piglets with IUGR

Intestinal mucus layer, intestinal epithelial cells, immune cells, normal microorganisms, and their metabolites in the intestinal lumen constitute the intestinal barrier of animals, and the dynamic balance between the barriers is the key to maintaining the homeostasis of the intestinal environment [10,11]. As a key composition of the intestinal mucosal epithelial barrier, ZO-1, claudin-1, and occludin are closely related to intestinal permeability [10]. In the present study, the IC group had lower *ZO-1*, *Claudin-1*, and *Occludin* gene expressions, which is consistent with previous studies [4,42]. EGF is an important regulatory factor of intestinal mucosal homeostasis and has a strong repair effect on intestinal morphological damage caused by weaning [43], intestinal ischemia/reperfusion [22], and necrotizing enterocolitis [44]. Similarly, the results of this study also indicated that EGF could promote the intestinal barrier function of piglets suffered from IUGR by increasing the expressions of *ZO-1*, *Claudin-1,* and *Occludin* genes. 

The mucus layer provides the first defense line of the gastrointestinal tract, which can effectively prevent the colonization of pathogenic microorganisms [45]. Mucins (MUCs), mainly MUC2, forms the bulk of the intestinal mucus, which can not only lubricate the intestine and antagonize pathogenic bacteria, but also participate in intercellular signal transduction and regulation of intestinal mucosal related immune factors [10,46]. EGF could repair intestinal mucosal injury by promoting intestinal MUC2 secretion [28,44,47]. Similarly, in the present study, the addition of EGF in diet significantly increased the gene expression of *MUC2* in the jejunum mucosa of IUGR piglets, which suggested that EGF can repair the intestinal barrier function damage caused by IUGR by promoting mucin secretion. 

The gut immune system provides an important barrier to prevent pathogens from penetrating the intestinal epithelium [11]. It was reported that IUGR could result in intestinal inflammatory injury, which was manifested by the increased secretion of proinflammatory cytokines [4,41] and the decreased secretion of sIgA [4]. Our present study showed that the IC group had a lower sIgA level and higher IL-1β, IL-6, and TNF-α levels compared with the NC group, which also suggested that IUGR caused intestinal inflammatory injury. EGF can promote intestinal immune function and alleviate intestinal inflammation by promoting the secretion of immunoglobulins and reducing the expression of proinflammatory factors [20,33,46]. For example, Chen et al. [20] showed that EGF treatment effectively reduced alcohol-induced inflammatory factors (IL-1β, IL-10, and TNF-α) in rats. Similarly, our present study showed that dietary EGF supplementation significantly increased the sIgA level and tended to decrease the IL-1β, IL-6, and TNF-α levels of IUGR piglets, which means that EGF alleviated the inflammatory injury caused by IUGR.

IUGR disrupts intestinal barrier function, while EGF provides protection for the intestinal barrier function of animals. It is generally believed that the integrity of intestinal barrier function is the basis of intestinal nutrients absorption, which determines the intestinal health and animal growth and development [11,48]. Pearson’s correlation analysis of the present study showed that indicators related to mechanical barrier (*ZO-1*, *Occludin* and *Claudin-1*), an indicator related to chemical barrier (*MUC2*), and an indicator related to immune barrier (sIgA) were positively correlated with the growth performance, glucose absorption, and intestinal morphology of piglets; and proinflammatory cytokines (IL-1β, IL-6, TNF-α) were negatively correlated with the growth performance, glucose absorption capacity, and intestinal morphology of piglets. These results suggest that EGF can promote intestinal development and nutrient absorption by promoting intestinal barrier function, thus improving the growth performance of IUGR piglets.

## 5. Conclusions

In summary, EGF has a positive effect on the growth performance and serum biochemical indexes of IUGR piglets; meanwhile, EGF could alleviate the intestinal injury of piglets caused by IUGR, which is indicated by the better intestinal morphology, enhanced intestinal glucose absorption capacity and antioxidant capacity, the improved intestinal barrier function, and the decreased intestinal inflammatory injury.

## Figures and Tables

**Table 1:** Composition and nutrient levels of the basal diet.

Ingredients ^1^	Content (%)	Item	Nutrient Levels ^3^
Corn	57.30	DE/(MJ/Kg)	14.08
Squeezed soybean meal	14.20	Crude protein (%)	20.14
Expanded soybean	13.53	Total Lys (%)	1.50
Whey powder	5.03	Total Thr (%)	0.75
Fish meal	4.60	Total Met (%)	0.82
Glucose	2.00	Met + Cys (%)	0.73
Limestone	0.84	Calcium (%)	0.86
CaHPO4	0.81	Available Pi (%)	0.45
L-lysine-HCl (98.0%)	0.34		
L-methionine (98.0%)	0.11		
NaCl	0.24		
Premix ^2^	1.00		
Total	100.00		

^1^ Corn (8.5% CP), squeezed soybean meal (47.9% CP), expanded soybean (35.5% CP), fish meal (67.0% CP), whey powder (11.55% CP). ^2^ The premix provided the following per kilogram of diet: VA, 10,000 IU; VB1, 1.8 mg; VB2, 5 mg; VB6, 7 mg; VB12, 0.02 mg; VD3, 1500 IU; VE, 60 IU; VK3, 3 mg; choline chloride, 1000 mg; niacin, 30 mg; pantothenic acid, 15 mg; biotin, 4.5 mg; folic acid, 0.3 mg; Cu, 150 mg; Zn, 125 mg; Fe, 120 mg; Mn, 5 mg; I, 0.3 mg; Se, 0.3 mg. ^3^ Crude protein and calcium levels were measured values, and other nutrient levels were calculated values.

**Table 2:** Primers used for RT-PCR.

Gene	Primers Sequence	Product Length	Reference
*AMPK-α1*	F:5′-GGTGAAAATCGGCCACTACA-3′ R:5′-TTGCCAACCTTCACTTTGCC-3′	72 bp	[23]
*SGLT1*	F:5′-ATATGCCCTTATATTCCCCTT-3′ R:5′-AAATCGTGTTGATAGCGCCAA-3′	138 bp	[23]
*GLUT2*	F:5′-CAGCCTATTCTAGTAGCACTG-3′ R:5′-AAATCGTGTTGATAGCGCCAA-3′	151 bp	[23]
*MUC2*	F: 5′-ACGCCATCCTGGGTGAGCT-3′ R: 5′-ACGCTGCCGTCCGACTTGA-3′	121 bp	[28]
*ZO-1*	F:5′-TGCTGGCACTGACCAACGTA-3′ R:5′-CACTGGGCATAATTCAGACGA-3′	129 bp	[4]
*Claudin-1*	F: 5′-TCCTGCTGGGACTAATAGCCAT-3′ R: 5′-CAATGACAGCCATCCGCATC-3′	102 bp	[4]
*Occludin*	F: 5′-CATTGCCATTGTACTAGGGTT-3′ R: 5′-GCTGCTCGTCATAAATACGTT-3′	140 bp	[4]
*β-actin*	F: 5′-CATCCTGCGTCTGGACCTGG-3′ R:5′-TAATGTCACGCACGATTTCC-3′	116 bp	[16]

**Table 3:** Effects of dietary EGF supplementation on the growth performance of piglets with IUGR.

Items	NC	IC	IE	SEM	*p* Value
IBW (kg)	6.77 ^a^	5.02 ^b^	5.04 ^b^	0.20	<0.001
FBW (kg)	9.74 ^a^	6.95 ^c^	7.63 ^b^	0.30	<0.001
ADG (g/d)	212.14 ^a^	137.14 ^c^	184.88 ^b^	8.25	<0.001
ADFI (g/d)	301.71 ^a^	216.13 ^c^	268.47 ^b^	10.30	<0.001
F/G (g/g)	1.42	1.60	1.46	0.05	0.297

Values are expressed as means ± SEM, *n* = 6 (one pig in the IC group was dead, *n* = 5 in the IC group); NC: NBW piglets fed with basal diet; IC: IUGR piglets fed with basal diet; IE: IUGR piglets fed with basal diet supplemented with 2 mg/kg EGF. ^a,b,c^ means that values within a row with different superscript letters were significantly different. *p* < 0.05 was taken to indicate statistical significance.

**Table 4:** Effects of EGF on serum biochemical profiles of piglets with IUGR.

Items	NC	IC	IE	SEM	*p*-Value
ALT(U/L)	50.28	64.16	62.30	3.48	0.214
AST (U/L)	110.88 ^c^	163.31 ^a^	137.78 ^ab^	7.86	0.015
TG (mmol/L)	0.57	0.43	0.48	0.03	0.168
TP (g/L)	44.19 ^a^	33.10 ^b^	41.31 ^a^	1.83	0.032
BUN (mmol/L)	3.87	3.21	4.28	0.27	0.294
GLU (mmol/L)	4.29 ^a^	2.30 ^b^	3.91 ^a^	0.29	0.005
IgA (g/L)	1.23	0.96	1.14	0.08	0.368
IgG (g/L)	2.83 ^a^	1.76 ^b^	2.54 ^a^	0.16	0.011
IgM (g/L)	0.49 ^a^	0.34 ^b^	0.40 ^ab^	0.02	0.025

Values are expressed as means ± SEM, *n* = 6 (one pig in the IC group was dead, *n* = 5 in the IC group); NC: NBW piglets fed with basal diet; IC: IUGR piglets fed with basal diet; IE: IUGR piglets fed with basal diet supplemented with 2 mg/kg EGF. ^a,b,c^ means that values within a row with different superscript letters were significantly different. *p* < 0.05 was taken to indicate statistical significance.

## Data Availability

The data presented in this study are available on request from the corresponding author.

## References

[1] Wu G., Bazer F.W., Wallace J.M., Spencer T.E. (2006). Board-invited review: Intrauterine growth retardation: Implications for the animal sciences. J. Anim. Sci..

[2] Wang J., Chen L., Li D., Yin Y., Wang X., Li P., Dangott L.J., Hu W., Wu G. (2008). Intrauterine growth restriction affects the proteomes of the small intestine, liver, and skeletal muscle in newborn pigs. J. Nutr..

[3] Xiong L., You J., Zhang W., Zhu Q., Blachier F., Yin Y., Kong X. (2020). Intrauterine growth restriction alters growth performance, plasma hormones, and small intestinal microbial communities in growing-finishing pigs. J. Anim. Sci. Biotechnol..

[4] Tang X., Xiong K. (2022). Intrauterine Growth Retardation Affects Intestinal Health of Suckling Piglets via Altering Intestinal Antioxidant Capacity, Glucose Uptake, Tight Junction, and Immune Responses. Oxid. Med. Cell. Longev..

[5] Fung C.M., White J.R., Brown A.S., Gong H., Weitkamp J.H., Frey M.R., McElroy S.J. (2016). Intrauterine growth restriction alters mouse intestinal architecture during development. PLoS ONE.

[6] Matheson S.M., Walling G.A., Edwards S.A. (2018). Genetic selection against intrauterine growth retardation in piglets: A problem at the piglet level with a solution at the sow level. Genet. Sel. Evol..

[7] Zhu Y., Li T., Huang S., Wang W., Dai Z., Feng C., Wu G., Wang J. (2018). Maternal L-glutamine supplementation during late gestation alleviates intrauterine growth restriction-induced intestinal dysfunction in piglets. Amino Acids.

[8] Zhang H., Chen Y., Li Y., Wang T. (2020). Protective Effect of Polydatin on Jejunal Mucosal Integrity, Redox Status, Inflammatory Response, and Mitochondrial Function in Intrauterine Growth-Retarded Weanling Piglets. Oxid. Med. Cell. Longev..

[9] Che L., Zhou Q., Liu Y., Hu L., Peng X., Wu C., Zhang R., Tang J., Wu F., Fang Z. (2019). Flaxseed oil supplementation improves intestinal function and immunity, associated with altered intestinal microbiome and fatty acid profile in pigs with intrauterine growth retardation. Food Funct..

[10] Tang X., Liu H., Yang S., Li Z., Zhong J., Fang R. (2016). Epidermal Growth Factor and Intestinal Barrier Function. Mediators Inflamm..

[11] Tang X., Liu X., Zhong J., Fang R. (2021). Potential Application of *Lonicera japonica* Extracts in Animal Production: From the Perspective of Intestinal Health. Front. Microbiol..

[12] Olszewski J., Zabielski R., Skrzypek T., Matyba P., Wierzbicka M., Adamski A., Grzesiuk E., Sady M., Gajewski Z., Ferenc K. (2021). Differences in Intestinal Barrier Development between Intrauterine Growth Restricted and Normal Birth Weight Piglets. Animals.

[13] Chen Y., Zhang H., Chen Y., Jia P., Ji S., Zhang Y., Wang T. (2021). Resveratrol and its derivative pterostilbene ameliorate intestine injury in intrauterine growth-retarded weanling piglets by modulating redox status and gut microbiota. J. Anim. Sci. Biotechnol..

[14] Tang X., Xiong K., Wassie T., Wu X. (2022). Curcumin and Intestinal Oxidative Stress of Pigs with Intrauterine Growth Retardation: A Review. Front. Nutr..

[15] Wang L., Zhu F., Yang H., Li J., Li Y., Ding X., Xiong X., Ji F., Zhou H., Yin Y. (2020). Epidermal growth factor improves intestinal morphology by stimulating proliferation and differentiation of enterocytes and mTOR signaling pathway in weaning piglets. Sci. China Life Sci..

[16] Tang X., Liu B., Wang X., Yu Q., Fang R. (2018). Epidermal growth factor, through alleviating oxidative stress, protect IPEC-J2 cells from lipopolysaccharides-induced apoptosis. Int. J. Mol. Sci..

[17] Xue J., Xie L., Liu B., Zhou L., Hu Y., Ajuwon K.M., Fang R. (2021). Dietary Supplementation of EGF Ameliorates the Negatively Effects of LPS on Early-Weaning Piglets: From Views of Growth Performance, Nutrient Digestibility, Microelement Absorption and Possible Mechanisms. Animals.

[18] Tang X., Xiong K. (2021). Effects of epidermal growth factor on glutamine and glucose absorption by IPEC-J2 cells challenged by lipopolysaccharide using the Ussing chamber system. Pak. J. Zool..

[19] Guntaka S.R., Samak G., Seth A., LaRusso N.F., Rao R. (2011). Epidermal growth factor protects the apical junctional complexes from hydrogen peroxide in bile duct epithelium. Lab. Investig..

[20] Chen Y.L., Peng H.C., Hsieh Y.C., Yang S.C. (2014). Epidermal growth factor improved alcohol-induced inflammation in rats. Alcohol.

[21] Suzuki T., Seth A., Rao R. (2008). Role of phospholipase Cγ-induced activation of protein kinase Cϵ (PKCϵ) and PKCβ1 in epidermal growth factor-mediated protection of tight junctions from acetaldehyde in Caco-2 cell monolayers. J. Biol. Chem..

[22] Arda-Pirincci P., Bolkent S. (2014). The role of epidermal growth factor in prevention of oxidative injury and apoptosis induced by intestinal ischemia/reperfusion in rats. Acta Histoch..

[23] Tang X., Xiong K. (2022). Epidermal growth factor activates EGFR/AMPK signalling to up-regulate the expression of SGLT1 and GLUT2 to promote intestinal glucose absorption in lipopolysaccharide challenged IPEC-J2 cells and piglets. Ital. J. Anim. Sci..

[24] NRC (2012). Nutrient Requirements of Swine.

[25] Tang X., Liu X., Zhang K. (2021). Effects of Microbial Fermented Feed on Serum Biochemical Profile, Carcass Traits, Meat Amino Acid and Fatty Acid Profile, and Gut Microbiome Composition of Finishing Pigs. Front. Vet. Sci..

[26] Peng P., Deng D., Chen S., Li C., Luo J., Romeo A., Li T., Tang X., Fang R. (2020). The effects of dietary porous zinc oxide supplementation on growth performance, inflammatory cytokines and tight junction’s gene expression in early-weaned piglets. J. Nutr. Sci. Vitaminol..

[27] Tang X., Su W., Fang R. (2019). Effects of calcitonin on porcine intestinal epithelial cells proliferation, phosphorus absorption, and NaPi-IIb expression. Pak. J. Zool..

[28] Wang L.X., Zhu F., Li J.Z., Li Y.L., Ding X.Q., Yin J., Xiong X., Yang H.S. (2020). Epidermal growth factor promotes intestinal secretory cell differentiation in weaning piglets via Wnt/β-catenin signalling. Animal.

[29] Wang X., Tan B., Liao P., Cui Z., Zhang S., Li X., Yin Y., Xiao D. (2020). Functional bioactive substance improves the growth performance, antioxidant capacity and immune function of growth retardation pigs. Food Agric. Immunol..

[30] Bai K., Jiang L., Li Q., Zhang J., Zhang L., Wang T. (2021). Dietary dimethylglycine sodium salt supplementation improves growth performance, redox status, and skeletal muscle function of intrauterine growth-restricted weaned piglets. J. Anim. Sci..

[31] Bedford A., Huynh E., Fu M., Zhu C., Wey D., de Lange C., Li J. (2014). Growth performance of early-weaned pigs is enhanced by feeding epidermal growth factor-expressing Lactococcus lactis fermentation product. J. Biotechnol..

[32] Carobene A., Braga F., Roraas T., Sandberg S., Bartlett W.A. (2013). A systematic review of data on biological variation for alanine aminotransferase, aspartate aminotransferase and γ-glutamyl transferase. Clin. Chem. Lab. Med..

[33] Morrill J.L., Morrill J.M., Feyerherm A.M., Laster J.F. (1995). Plasma proteins and a probiotic as ingredients in milk replacer. J. Dairy Sci..

[34] Wang S., Guo C., Zhou L., Zhong Z., Zhu W., Huang Y., Zhang Z., Gorgels T.G.M.F., Berendschot T.T.J.M. (2015). Effects of dietary supplementation with epidermal growth factor-expressing saccharomyces cerevisiae on duodenal development in weaned piglets. Brit. J. Nutr..

[35] Schroeder H.W., Cavacini L. (2010). Structure and function of immunoglobulins. J. Allergy Clin. Immunol. Pract..

[36] Zou L., Xiong X., Liu H., Zhou J., Liu Y., Yin Y. (2019). Effects of dietary lysozyme levels on growth performance, intestinal morphology, immunity response and microbiota community of growing pigs. J. Sci. Food Agric..

[37] Chen C., Yin Y., Tu Q., Yang H. (2018). Glucose and amino acid in enterocyte: Absorption, metabolism and maturation. Front. Biosci. (Landmark Ed).

[38] Chen L., Tuo B., Dong H. (2016). Regulation of Intestinal Glucose Absorption by Ion Channels and Transporters. Nutrients.

[39] Schmitt C.C., Aranias T., Viel T., Chateau D., Le Gall M., Waligora-Dupriet A.J., Melchior C., Rouxel O., Kapel N., Gourcerol G. (2016). Intestinal invalidation of the glucose transporter GLUT2 delays tissue distribution of glucose and reveals an unexpected role in gut homeostasis. Mol. Metab..

[40] Koepsell H. (2020). Glucose transporters in the small intestine in health and disease. Pflugers Arch..

[41] Pirinccioglu A.G., Gökalp D., Pirinccioglu M., Kizil G., Kizil M. (2010). Malondialdehyde (MDA) and protein carbonyl (PCO) levels as biomarkers of oxidative stress in subjects with familial hypercholesterolemia. Clin. Biochem..

[42] Yan E., Zhang J., Han H., Wu J., Gan Z., Wei C., Zhang L., Wang C., Wang T. (2019). Curcumin Alleviates IUGR Jejunum Damage by Increasing Antioxidant Capacity through Nrf2/Keap1 Pathway in Growing Pigs. Animals.

[43] Xu S., Wang D., Zhang P., Lin Y., Fang Z., Che L., Wu D. (2015). Oral administration of Lactococcus lactis-expressed recombinant porcine epidermal growth factor (rpEGF) stimulates the development and promotes the health of small intestines in early-weaned piglets. J. Appl. Microbiol..

[44] Clark J.A., Doelle S.M., Halpern M.D., Saunders T.A., Holubec H., Dvorak K., Boitano S.A., Dvorak B. (2006). Intestinal barrier failure during experimental necrotizing enterocolitis: Protective effect of EGF treatment. Am. J. Physiol. Gastrointest. Liver Physiol..

[45] Paone P., Cani P.D. (2020). Mucus barrier, mucins and gut microbiota: The expected slimy partners?. Gut.

[46] Pelaseyed T., Bergström J.H., Gustafsson J.K., Ermund A., Birchenough G.M., Schütte A., van der Post S., Svensson F., Rodríguez-Piñeiro A.M., Nyström E.E. (2014). The mucus and mucins of the goblet cells and enterocytes provide the first defense line of the gastrointestinal tract and interact with the immune system. Immunol. Rev..

[47] Bedford A., Chen T., Huynh E., Zhu C., Medeiros S., Wey D., de Lange C., Li J. (2015). Epidermal growth factor containing culture supernatant enhances intestine development of early-weaned pigs in vivo: Potential mechanisms involved. J. Biotechnol..

[48] Schoultz I., Keita Å.V. (2020). The Intestinal Barrier and Current Techniques for the Assessment of Gut Permeability. Cells.

